# Remimazolam in perioperative management of Eisenmenger syndrome: a case report

**DOI:** 10.1186/s40981-024-00692-6

**Published:** 2024-02-02

**Authors:** Kazuya Hashimoto, Tsuguhiro Matsumoto, Toshiyuki Mizota, Shinichi Kai, Moritoki Egi

**Affiliations:** Department of Anesthesia, Kyoto University Hospital, Kyoto, Japan

**Keywords:** Eisenmenger syndrome, Pulmonary hypertension, Remimazolam

## Abstract

**Background:**

Eisenmenger syndrome (ES) is characterized by severe and irreversible pulmonary hypertension stemming from an uncorrected intracardiac shunt of significant size. The imbalance between systemic and pulmonary artery pressures predisposes patients with ES to the risk of cardiac arrest. Remimazolam has caused less circulatory depression, which may be advantageous for ES. However, no studies reported the use of remimazolam in perioperative ES management.

**Case presentation:**

A 45-year-old female patient with ES derived from a ventricular septal defect was scheduled to undergo bilateral adnexectomy for an ovarian tumor. Her oxygen saturation was 80% with 3 L/min oxygen at rest, and her pulmonary and systemic flow ratio was 0.53. She underwent general anesthesia with remimazolam, and intraoperative hemodynamics was stable without hypotension or reduced oxygen saturation.

**Conclusions:**

Our successful management of ovarian tumor surgery in a patient with ES using remimazolam reveals its potential effectiveness in perioperative care.

## Background

Eisenmenger syndrome (ES) is characterized by severe and irreversible pulmonary hypertension caused by a large uncorrected intracardiac shunt [1]. The direction of shunt blood flow is based on a delicate balance between systemic and pulmonary artery pressure, and a sudden decrease in systemic artery blood pressure increases right-left shunting, causing hypoxemia, myocardial ischemia, and cardiac arrest [2, 3]. Remimazolam, a short-acting benzodiazepine, has been reported to maintain cardiac output (CO) and cause less hypotension than propofol [4, 5], making it useful for patients who may be hemodynamically unstable [6, 7]. Only one case report was presented on the anesthesia management of patients with pulmonary hypertension (PH) using remimazolam [6], and no case reports with ES have been published. Herein, we describe the successful perioperative management of open bilateral adnexectomy with remimazolam in a patient with ES.

## Case presentation

A 45-year-old female patient (164 cm, 52 kg) who visited her previous doctor 3 months ago with complaints of lower abdominal pain and lumps was diagnosed with an ovarian tumor. She was diagnosed with a ventricular septal defect (VSD) at an early age, and with ES at her school age. Medical therapy for ES began 9 years ago with oral tadalafil (phosphodiesterase 5 inhibitor) of 40 mg/day, macitentan (endothelin receptor antagonist) of 10 mg/day, and selexipag (prostacyclin receptor agonist) of 400 μg/day. Home oxygen therapy was introduced for hypoxemia. Thereafter, she led her daily life without problems, but she gradually began to have difficulty in her daily life after becoming aware of lower abdominal pain and lumps and even walking on level ground became difficult. The tumor was suspected to be ovarian cancer, and the patient expressed a strong desire for surgery due to accompanying symptoms. However, because of the presence of severe ES, multiple hospitals found perioperative management challenging, leading to a referral to our hospital.

Her oxygen saturation was 80% with 3 L/min oxygen at rest but dropped to 52% during a 6-min walk test. Transthoracic echocardiography revealed a ventricular septal defect with a bilateral shunt. The right ventricular wall was thickened to 10 mm, and the tricuspid regurgitation pressure gradient was 133 mmHg. There were no findings of decreased left and right ventricular contraction. Right heart catheterization results were as follows: pulmonary artery pressure (PAP) of 123/52 mmHg, arterial pressure of 115/69 mmHg, CO of 3.87 L/min, cardiac index (CI) of 2.44 L/min/m^2^, pulmonary and systemic flow ratio of 0.53, and pulmonary vascular resistance (PVR) of 28.94 Wood Units.

Anesthesiologists, gynecologists, and cardiologists held a joint preoperative conference to discuss her perioperative management and indicated for open bilateral adnexectomy for the ovarian tumor which could be performed under general anesthesia. First, a cardiologist placed 5-Fr and 7-Fr sheaths in her femoral artery and vein, respectively, before entering the operating room to allow the use of extracorporeal membrane oxygen to prevent intraoperative and postoperative cardiac arrest. Further, a pulmonary artery catheter was placed in her right internal jugular vein. Once in the operating room, the anesthesiologist inserted an arterial catheter and a central venous catheter into the right radial artery and left internal jugular vein, respectively. Noradrenaline of 0.1 μg/kg/min was then initiated before induction of anesthesia.

**Fig. 1:**
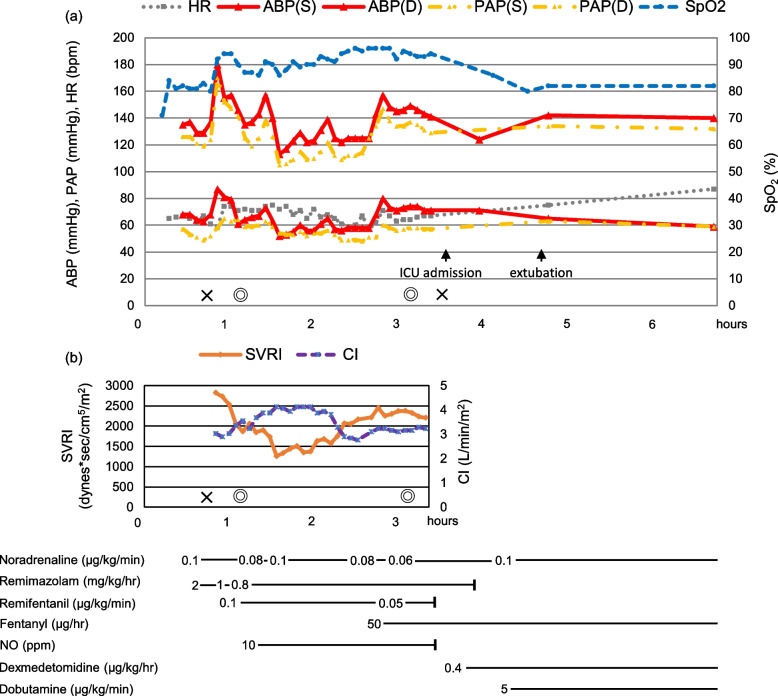
Intraoperative and postoperative record. **a** HR heart rate, ABP arterial blood pressure, (S) systolic, (D) diastolic, PAP pulmonary arterial pressure, SpO_2_ saturation of percutaneous oxygen. **b** SVRI systemic vascular resistance index, CI cardiac index

Perioperative records are shown in Fig. 1. Anesthesia was induced with remimazolam of 2 mg/kg/h, fentanyl of 250 μg, and rocuronium of 50 mg, and maintained with remimazolam of 0.8 mg/kg/h and remifentanil of 0.1 μg/kg/min. The tracheal intubation was performed after 4 ml of 4% lidocaine was sprayed into the trachea. In addition to standard monitoring, invasive arterial blood pressure, PAP, central venous pressure, bispectral index (BIS), cerebral regional oxygen saturation (rSO_2_), CO/CI/systemic vascular resistance index (SVRI) using the FloTrac system, and transesophageal echocardiography were monitored during surgery. Intraoperative systolic PAP remained between 100 and 140 mmHg, but rarely exceeded the systolic artery pressure and SpO_2_ always remained > 88% at a fraction of inspiration O_2_ of 50% (Fig. 1a). SVRI was 2825 Dynes × s/cm^5^/m^2^ before induction of anesthesia, but once dropped to 1259 Dynes × s/cm^5^/m^2^ after laparotomy and then rose and remained between 1500 and 2300 Dynes × s/cm^5^/m^2^ (Fig. 1b). Intraoperative nitric oxide of 10 ppm administration was attempted, but the hemodynamics and SpO_2_ were unchanged. BIS was 37–62 and rSO2 was 59–77% during general anesthesia. Bilateral transversus abdominis fascia plane blocks were performed after surgical completion, and the patient was transferred to the intensive care unit (ICU) under sedation with tracheal intubation. Anesthesia time was 2 h and 45 min, operation time was 1 h and 53 min, and blood loss was 90 ml. Remimazolam of 0.8 mg/kg/h was continued after ICU admission and was discontinued 30 min thereafter. Dexmedetomidine was initiated at 0.4 μg/kg/h upon ICU admission. Fentanyl of 50 μg/h was administered for postoperative analgesia. Bradycardia, hypotension, and decreased SpO2 were observed with arousal 50 min after admission; therefore, dobutamine of 5 μg/kg/min was initiated, followed by a gradual improvement in hemodynamics and oxygenation. The tracheal tube was removed 1 h and 40 min after admission and fitted with a high-flow nasal cannula. After extubation, SpO_2_ was maintained at approximately 80%. She was transferred to the general ward on postoperative day (POD) 3 and discharged on POD 11 without any complications. The patient signed written informed consent for this case report.

## Discussion

ES represents the most severe phenotype of pulmonary artery hypertension associated with congenital heart disease (CHD) and occurs in patients with large unrepaired shunts such as VSD [1]. The perioperative mortality rate for noncardiac surgery in patients with ES was once reported as 23.5% [8], but the mortality rate was 3.8% in a recent case series of 53 cases [3]. The authors reported it reflected the immediacy of expert consultation available in a tertiary-care academic center and increasing knowledge related to management of adults with CHD using a multidisciplinary approach [3]. In this case, the patient was managed safely as a result of a thorough discussion about perioperative management and appropriate backup arrangements.

While there are no evidence-based guidelines about perioperative management of patients with ES, many studies have reported the importance of avoiding hypotension and elevation of PAP to prevent an increase in right-to-left shunting [1–3]. The selection of drugs with minimal circulatory depression, tight anesthetic titration, and the appropriate use of vasopressors is recommended to avoid hypotension [2, 3]. Hypoxemia, hypercarbia, and acidosis must be corrected to avoid PAP elevation. In this case, we first opted for general anesthesia because spinal or epidural anesthesia can cause rapid hypotension and strict respiratory control is difficult. Second, noradrenaline was administered before induction of anesthesia to prevent associated hypotension. Third, the patient was anesthetized with total intravenous anesthesia without inhaled anesthetics, considering that the VSD shunt flow, in this case, was bidirectional and the pulmonary blood flow was not constant. We selected remimazolam, among intravenous anesthetics, because of its weak circulatory depressant effect [6, 7].

Remimazolam is a short-acting benzodiazepine with pharmacological properties of rapid onset, fast recovery, and relatively small effects on hemodynamics [4]. Therefore, it has been used in a variety of settings, including open heart surgery [9], transcatheter aortic valve replacement [10], liver transplantation [7], and anesthesia for patients with PH [6]. A randomized controlled trial (RCT) comparing the use of remimazolam and propofol during induction of anesthesia reported significantly higher blood pressure and CO in the remimazolam group, and the incidence of hypotension was 3% in the remimazolam group compared with 63% in the propofol group [4]. Another RCT similarly revealed a significantly lower incidence of hypotension in the group using remimazolam for anesthesia induction compared with propofol [5]. Additionally, a case report using remimazolam in a patient with PH revealed that remimazolam had little effect on PVR [6], and animal studies in rats have reported that midazolam, a benzodiazepine like remimazolam, has little effect on PVR [11]. In this case, hemodynamics were very stable during induction and maintenance of anesthesia, with no hypotension requiring bolus administration of vasopressors, no decrease in CI, and no significant increase in PAP.

In conclusion, we have safely managed ovarian tumor surgery in a patient with ES using remimazolam. We believe that remimazolam may be an effective option for the anesthetic management of patients with ES.

## Data Availability

All data are included in this article.
